# JMJD8 overexpression in breast cancer: implications for diagnosis, prognosis, and immune microenvironment interactions

**DOI:** 10.3389/fonc.2025.1536278

**Published:** 2025-07-21

**Authors:** Chenlei Zhu, Tianyi Xi, Guorong Yang, Wen Lu, Sentai Wang, Jiwei Cao

**Affiliations:** Department of General Surgery, The Affiliated Suzhou Hospital of Nanjing Medical University, Suzhou Municipal Hospital, Gusu School, Nanjing Medical University, Suzhou, China

**Keywords:** JMJD8, breast cancer, overall survival, IFN-β, immune microenvironment

## Abstract

**Background:**

Breast cancer has severe consequences due to late diagnosis and the lack of effective therapies. Currently, potential biomarkers for breast cancer have not been systematically evaluated. Research has shown that JMJD8 is associated with cGAS-STING pathway and plays a role in various tumor microenvironments, but its relationship with breast cancer remains unclear. We investigate the relationship between JMJD8 and the prognosis and immune infiltration microenvironment of breast cancer, exploring its potential as a prognostic biomarker for this type of cancer.

**Methods:**

In this study, we utilized data from The Cancer Genome Atlas (TCGA) to assess the association between JMJD8 expression and clinical characteristics in breast cancer (BRCA) patients through the Wilcoxon signed-rank test and logistic regression. Additionally, we employed Kaplan-Meier and Cox regression methods to confirm the impact of JMJD8 expression levels on overall survival. We constructed JMJD8 knockout BRCA cell lines and studied the effects of JMJD8 protein on tumor cell proliferation and anti-tumor immunity at both cellular and animal levels.

**Results:**

Compared to normal tissues, JMJD8 expression levels were significantly elevated in BRCA tissues. High JMJD8 expression was closely associated with advanced pathological stages and was identified as an independent factor negatively impacting overall survival. In both cellular and animal experiments, JMJD8 knockout relieved the inhibition of the cGAS-STING pathway. This resulted in a significant enhancement of the anti-tumor immune response, as it induced dendritic cell (DC) antigen presentation and maturation, ultimately inhibiting the proliferation of BRCA cells. Furthermore, the JMJD8 expression was positively correlated with the infiltration of M2 macrophages in the tumor microenvironment, suggesting that JMJD8 may contribute to the deterioration of the tumor immunosuppressive microenvironment, potentially leading to reduced patient survival.

**Conclusion:**

The elevated expression of JMJD8 in breast cancer tissues is indicative of its involvement in the progression of the disease and its association with immune cell infiltration patterns. Our findings support the hypothesis that JMJD8 could serve as a prognostic biomarker, reflecting the immunosuppressive characteristics of the tumor microenvironment and aiding in the development of targeted therapeutic strategies for breast cancer management.

## 1 Introduction

The Jumonji C (JmjC) domain-containing protein family has been shown to catalyze site-specific demethylation of histone and non-histone proteins. This catalytic process utilizes an oxidative mechanism that requires Fe (II) and α-ketoglutarate (αKG) as cofactors. As a result, this demethylation leads to the formation of demethylation products and formaldehyde (1). JMJD8, as a member of the JMJD family, is characterized by its relatively low molecular weight; however, its specific function has not yet been clearly elucidated (2). While JMJD8 does not display the anticipated enzymatic activity due to substitutions at the residues of its demethylation active sites, its distinctive endoplasmic reticulum-anchoring mechanism enables its participation in various physiological processes, such as protein folding, protein aggregation, DNA repair, and tumor immunity (3–6).

JMJD8 expression is strongly associated with tumor progression. In non-small cell lung cancer (NSCLC), JMJD8 enhances the proliferation and invasion of cancer cells by stabilizing the epidermal growth factor receptor (EGFR), thereby inhibiting the degradation of the overexpressed EGFR (7). In squamous cell carcinoma, the knockdown of JMJD8 significantly inhibits the invasive growth of tumors (8). Recent research has demonstrated that JMJD8 functions as a suppressor of STING-induced type I interferons. By competing with TBK1 for binding to the C-terminal peptide of STING, JMJD8 inhibits the induction of type I interferons and interferon-stimulated genes (ISGs) through the cGAS-STING pathway, thereby influencing immune cell infiltration (9). Additionally, JMJD8 has garnered attention for its participation in the TNF-mediated NF-κB pathway (4) and endogenous inflammatory pathways (10). This involvement has heightened our interest in understanding its role in tumor immunity.

While existing studies have characterized aberrant JMJD8 overexpression in neoplastic tissues and its association with clinicopathological parameters across diverse malignancies (11), the multifaceted contribution of JMJD8 to BRCA pathogenesis, notably its mechanistic regulation of the tumor immune microenvironment and functional involvement in immune checkpoint-driven evasion pathways, remains to be fully elucidated. Globally, BRCA was the most commonly diagnosed cancer in 2020, with over two million new cases, accounting for approximately 11% of all diagnosed cancers. In terms of mortality, it ranked fifth, with nearly 700,000 deaths, representing around 7% of all cancer-related deaths (12). The intricate immunosuppressive microenvironment significantly influences the initiation, progression, and invasion of breast cancer (13, 14). This study aims to evaluate whether JMJD8 expression levels could serve as potential diagnostic and prognostic biomarkers, and to explore its association with immune microenvironment in BRCA.

## 2 Materials and methods

### 2.1 Dataset source and preprocessing

The expression levels of JMJD8 in 113 BRCA samples, along with their adjacent normal tissues, were analyzed using mRNA sequencing data from The Cancer Genome Atlas (TCGA-BRCA) dataset. Clinical characteristics, including tumor status, histologic grade, pathologic stage, and vascular invasion, were assessed in relation to their respective clinical patterns. Differences in transcriptional expression were evaluated using Student’s T-test.

### 2.2 Immunohistochemical staining

We further investigated JMJD8 protein levels in BRCA through immunohistochemistry (IHC) on a commercial tissue microarray (Bioaitech Co., Xi’an, China, cat # F601101) comprising 10 BRCA and 10 normal tissue samples. The IHC procedure involved dewaxing and hydrating the tissues, followed by antigen retrieval and blocking endogenous peroxidase activity. Slides were then incubated with the primary antibody overnight at 4°C. After secondary antibody incubation, staining was performed using diaminobenzidine (DAB).

Two independent pathologists evaluated and scored the staining results. JMJD8 protein expression was quantified based on the proportion of positively stained tumor cells and staining intensity. Specifically, the percentage of immunoreactive tumor cells was scored as follows: 1 (<10%), 2 (10–25%), 3 (26–49%), and 4 (≥50%). Staining intensity was visually graded as 1 (negative), 2 (light yellow), 3 (light brown), and 4 (dark brown). The final immunoreactivity score for each sample was calculated by multiplying the percentage score by the intensity score.

### 2.3 Immune infiltration algorithm

Based on the ssGSEA algorithm provided in R-packet-GSVA [1.46.0], the provided markers of 22 immune cells were used to calculate the immune infiltration of the corresponding data.

### 2.4 Diagnostic analysis

The Receiver Operating Characteristic (ROC) curve was applied to assess the specificity and sensitivity of gene prediction accuracy, using the area under the ROC curve (AUC) as a diagnostic value based on the “pROC” package in the statistical software (version 1.18.0) was used.

### 2.5 Survival analysis

The Kaplan-Meier curve was performed to compare overall survival (OS) between the differential expression groups of JMJD8 including 1005 BRCA samples in the TCGA database and KM Plotter, respectively. The correlation between JMJD8 expression and survival was analyzed to discover significant prognostic factors.

### 2.6 Correlation analysis

Gene expression correlation analysis was performed for given sets of mRNA expression data in TCGA-BRCA were used for analysis. The correlation coefficient was determined by the Spearman method.

### 2.7 Cell culture

The breast cancer cell lines EMT6, 4T1, and the macrophage cell line RAW264.7 used in this study were obtained from the China Center for Type Culture Collection. The complete culture medium for cell growth consisted of Dulbecco’s Modified Eagle’s Medium (DMEM) supplemented with 10% fetal bovine serum (FBS) and 1% penicillin-streptomycin. Cells were cultured in an incubator at 37°C with 5% CO_2_, and those in the logarithmic growth phase were used for subsequent experiments.

### 2.8 Transfection of small interfering RNA

The siRNAs used in this study were sourced from Genscript Co., Ltd. (Nanjing, China). For siRNA transfection into breast cancer cell lines, we used Lipofectamine 2000 (Invitrogen, Carlsbad, USA), following the manufacturer’s protocol.

The transfection process began by seeding breast cancer cells in culture plates and growing them in an appropriate medium until they reached approximately 50% confluence. At this point, the siRNAs were prepared for transfection. Each siRNA was diluted in Opti-MEM medium and then mixed with Lipofectamine 2000 reagent in a separate tube. This siRNA-Lipofectamine 2000 mixture was incubated at room temperature for 20 minutes to allow siRNA-lipid complexes to form.

After incubation, the siRNA-lipid complex was added to the cells in the culture plates at a final siRNA concentration of 100 nM. The cells were then incubated with the transfection mixture for 48 hours to ensure efficient uptake and effective gene silencing.

The siRNAs used in this study had the following sequences:

siJMJD8-1: 5’-GACTTGCCCTTCCAGGAGT-3’

siJMJD8-2: 5’-GGCAATGACACCCTGTACT-3’

### 2.9 Quantitative real-time polymerase chain reaction

cDNA was amplified using SYBR-Green PCR Master Mix (TAKARA, Tokyo, Japan) on a QuantStudio 5 system (ABI, Carlsbad, USA) under the following conditions: initial denaturation at 95°C for 10 minutes, followed by 40 cycles of denaturation at 95°C for 15 seconds and annealing/extension at 60°C for 1 minute. JMJD8 mRNA levels were quantified using the 2^−ΔΔCt^ method, with GAPDH serving as the internal control.

The sequences of the specific primers used for amplification were as follows:

GAPDH forward, 5’-AGATCCCTCCAAAATCAAGTGG-3’;

GAPDH reverse, 5’- GGCAGAGATGATGACCCTTTT-3’;

JMJD8 forward, 5’-TCTTCGGGGACAACAACTTC-3’;

JMJD8 reverse, 5’-TCAGGTGGGTAAAGGAACCA-3’.

### 2.10 Western blotting

Following 72 hours of siRNA transfection, cells were collected and lysed in RIPA buffer. Equal amounts of protein from each group were separated via 12% SDS-PAGE and transferred onto PVDF membranes. The membranes were blocked with 5% BSA at room temperature for 1 hour, then incubated with anti-JMJD8 antibody (Abclonal, A10476) followed by HRP-conjugated secondary antibody (Abcam, ab288151). Signal detection was conducted using ECL chemiluminescence reagents, with results recorded on a chemiluminescent imaging system. Afterward, the membranes were stripped, re-blocked, and sequentially incubated with anti-actin antibody (Abcam, ab6276) and HRP-conjugated secondary antibody (Abcam, ab205719), with ECL detection and imaging performed once more.

The impact of JMJD8 knockdown on cGAS-STING pathway proteins was assessed via Western blotting. EMT6 or 4T1 cell lines, in different treatment groups, were seeded into 6-well plates at a density of 2 × 10^5 cells per well. After attachment, RAW264.7 cells were added at a density of 5 × 10^5 cells per well for co-culture over 36 hours. To analyze cGAS-STING pathway activation, total protein was extracted from the co-cultured cells using a non-denaturing tissue/cell lysis kit (Solarbio) with a broad-spectrum protease inhibitor mixture (EDTA-free, BOSTER) and a broad-spectrum phosphatase inhibitor mixture (EDTA-free, BOSTER). For Western blot analysis, 40 μg of protein was used, following the protocol described above. The antibodies included Anti-β-actin (CST, 4967),Anti-IRF3 (CST, 4302), Anti-Phospho-IRF3 (CST, 4947), Anti-TBK1 (CST, 3504), Anti-Phospho-TBK1 (CST, 5483) And Anti PD-L1 (CST, 64988).

### 2.11 Subcutaneous tumor xenograft model of breast cancer cells

After 72 hours of transfection, the cells were collected for tumor xenograft implantation. The Breast Cancer xenograft model was established by subcutaneously injecting different groups of Breast Cancer cells into the right armpits of six-week-old BALB/c nude mice (1×10^7 cells per mouse; three animals per group). Tumor size was recorded every other day, and tumor volume was calculated according to the formula: width^2 × length/2. Fourteen days after tumor cell implantation, all mice were euthanized, and tumor tissues were collected.

### 2.12 *In vivo* immune cell infiltration assessment

To analyze the overall immune landscape of breast cancer, tumor-draining lymph nodes (TDLNs) and tumor tissues were harvested 14 days post-treatment. Fresh tissues were carefully dissected into small fragments and digested using a mixture of enzymes, including neutral protease, collagenase, and hyaluronidase, to prepare a single-cell suspension. The isolated cells were then stained with Alexa Fluor 488 anti-mouse CD11c, Alexa Fluor 647 anti-mouse CD86, PE anti-mouse CD80 antibodies, APC anti-mouse CD8a Antibody, PE/Cy5 anti-mouse CD3 Antibody and PE/Cy7 anti-mouse CCR7 Antibody (BioLegend, USA) before being analyzed by flow cytometry using a BD Calibur system. Additionally, the secretion of interferon-gamma (IFN-β) within the tumors was quantified using an ELISA kit (Dakewe Biotech Co., Ltd.).

### 2.13 Statistical analysis

The Wilcoxon rank sum test was used to assess the significant differential expression levels of the JMJD8 in BRCA with the threshold of gene expression being selected as the median method. Correlation of gene expression was evaluated by Spearman’s correlation coefficient. Univariate Cox analysis was used to screen for potential risk factors, and multivariate Cox analysis was used to verify the independent variate of JMJD8 expression on overall survival. All statistical analyses were performed with the R statistical software (version 4.2.1). A P-value of less than 0.05 is considered statistically significant.

## 3 Results

### 3.1 Expression of the JMJD8 in BRCA

**Figure 1:**
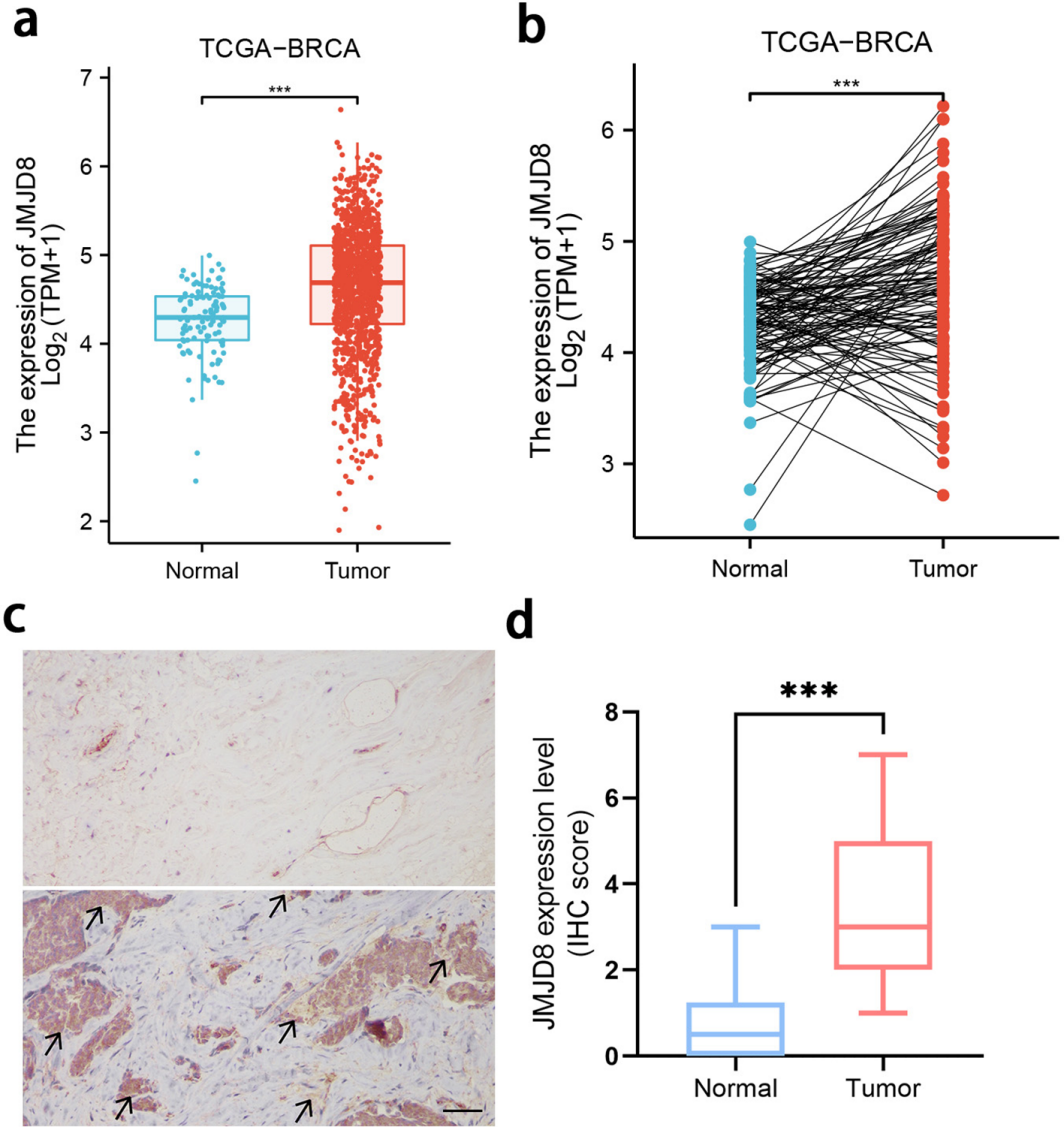
Expression of the JMJD8 in BRCA. Evaluation of JMJD8 expression in patients with BRCA compared to adjacent normal tissue samples: **(a)** unpaired samples, **(b)** paired samples; **(c)** Expression and **(d)** scoring of JMJD8 protein, as detected by IHC in 10 BRCA and 10 normal tissues (scale bar = 50 μm). ***p<0.001.

We conducted a comprehensive analysis of JMJD8 mRNA expression levels in BRCA samples alongside adjacent normal tissue samples, utilizing RNA-sequencing datasets from TCGA. Our findings revealed that JMJD8 was significantly upregulated in breast cancer tissues when compared to normal adjacent tissues (**Figures 1a, b**, **Supplementary Table S1**). Then, we examined JMJD8 protein levels in a tissue microarray consisting of 10 normal tissues and 10 BRCA tissues using IHC. The results showed that JMJD8 expression was significantly higher in BRCA tissues compared to normal tissues at the protein level (**Figures 1c, d**). This upregulation suggests a potential role for JMJD8 in the pathogenesis of breast cancer and highlights its importance as a candidate for further investigation in this context.

### 3.2 Correlation between JMJD8 expression and BRCA clinicopathological parameters

To further investigate the clinical implications of JMJD8 expression, we conducted a subgroup analysis of RNA-sequencing data from TCGA. Our results indicate that JMJD8 is significantly upregulated in breast cancer tissues characterized by advanced pathological stages and higher histological grades. Additionally, we found a notable increase in JMJD8 expression in patients with negative HER2 status and positive estrogen receptor (ER) status. This upregulation was also observed across different menopausal statuses, both pre- and post-menopause, and was particularly pronounced in the luminal A (lumA) subtype of breast cancer.

**Figure 2:**
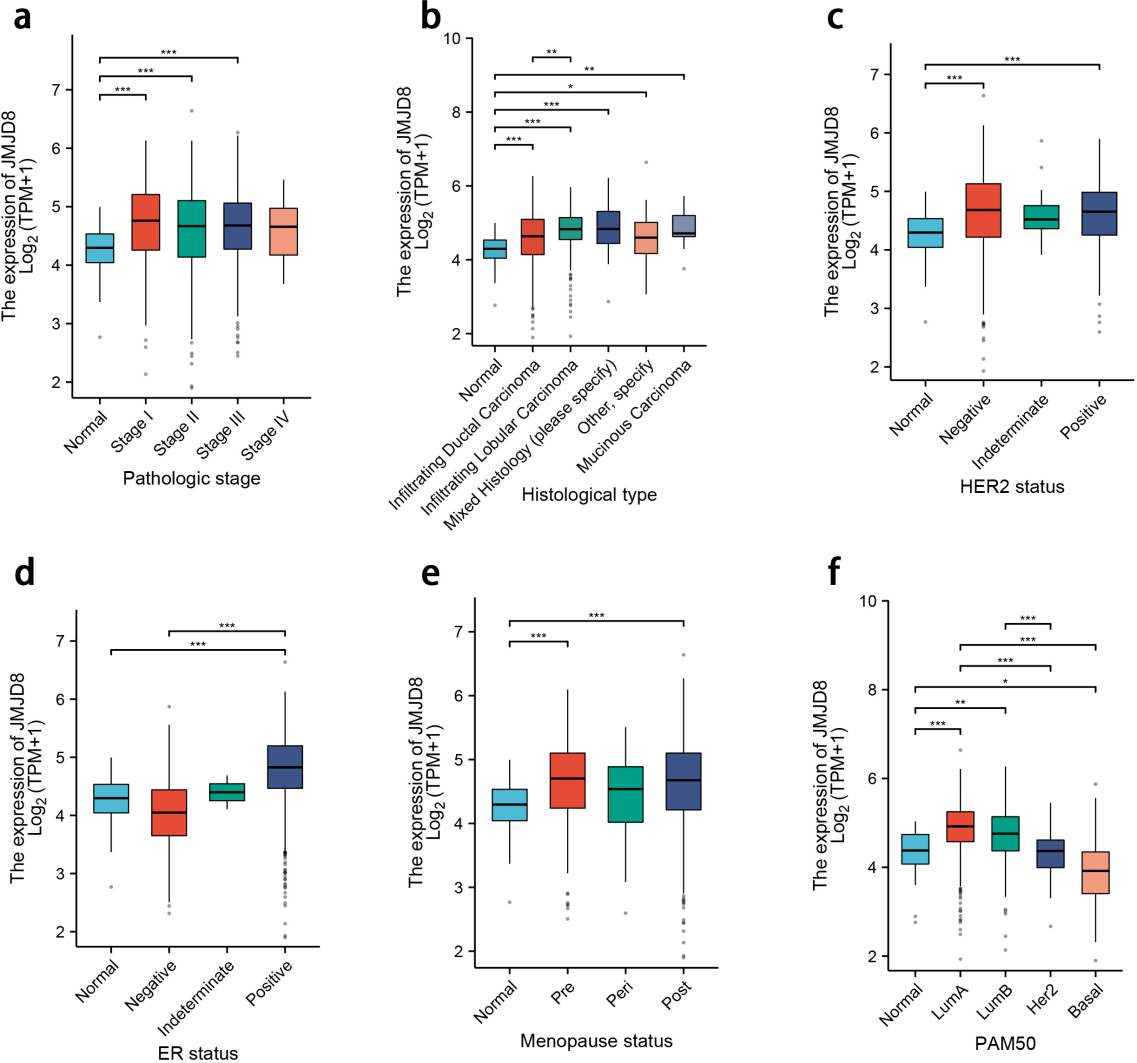
Differential expression levels of JMJD8 in subgroups with clinical features. **(a)** Pathologic stage; **(b)** Histological grades; **(c)** HER2 status; **(d)** ER status; **(e)** Menopause status; **(f)** PAM50 status. *p<0.05; **p<0.01; ***p<0.001.

These findings suggest that elevated JMJD8 expression is intricately linked to more aggressive clinical characteristics, highlighting its potential role as a biomarker for tumor progression and aggressiveness. The association of JMJD8 with key clinical features underscores its relevance in understanding breast cancer pathology and could inform future therapeutic strategies aimed at targeting this molecule (**Figure 2**).

### 3.3 Diagnostic value and prognostic potential of JMJD8 in BRCA

**Figure 3:**
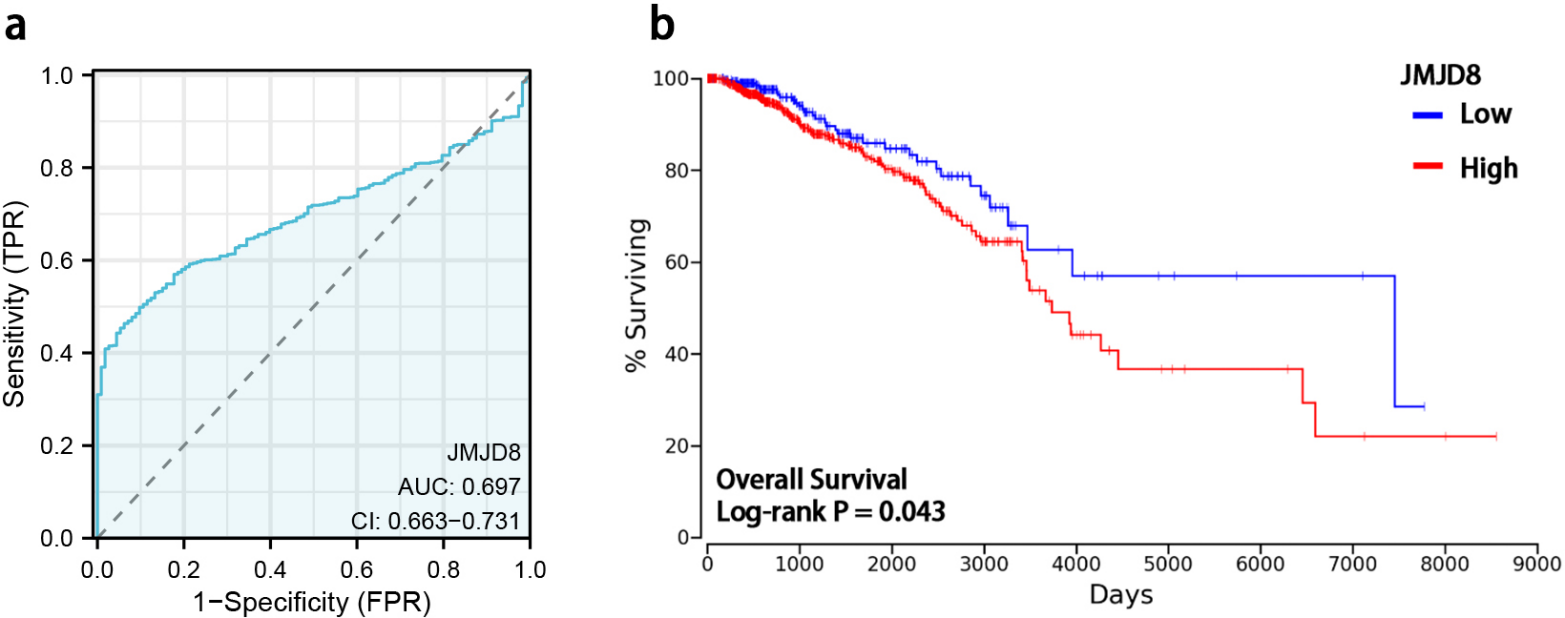
Diagnostic value and prognostic potential of JMJD8 in BRCA. **(a)** ROC curves and the predictability of JMJD8; **(b)** Kaplan-Meier curve of overall survival and expression levels of JMJD8.

We utilized ROC curves to evaluate the predictive performance of JMJD8 for BRCA outcomes. Our analysis indicated that JMJD8 demonstrates a notable predictive accuracy when distinguishing between normal and tumor tissue, with AUC of 0.697 (95% CI: 0.663–0.731), as illustrated in **Figure 3a**.

To further investigate the prognostic significance of JMJD8 mRNA expression levels in BRCA, we employed Kaplan-Meier survival analysis to correlate expression levels with patient survival and clinical follow-up duration. Log-rank tests were conducted to assess statistical significance. The results of our study revealed that elevated JMJD8 mRNA expression is significantly associated with poor prognosis (Log-rank p=0.043), as shown in **Figure 3b**. These findings underscore the potential of JMJD8 as a valuable biomarker for predicting clinical outcomes in breast cancer patients.

### 3.4 High-level expression of JMJD8 is an independent risk factor for OS of BRCA

Univariate Cox analysis showed that high JMJD8 expression was significantly correlated with poor Disease Specific Survival (HR=0.55 95%CI=0.30 – 0.99 P=0.046). (**Table 1**). Besides, Multivariate Cox analysis confirmed pathological stages (Stage III: HR=5.278, 95% CI=2.157 - 12.913, p<0.001; Stage IV: HR=30.386, 95% CI=11.130 - 82.960, p<0.001) were another independent risk factors in BRCA.

**Table 1:** Cox regression analyses of clinical characteristics related to BRCA Disease Specific Survival.

Characteristics	Total(N)	Univariate analysis		Multivariate analysis	
		Hazard ratio (95% CI)	P value	Hazard ratio (95% CI)	P value
Pathological stage	1,044				
Stage I	179	Reference		Reference	
Stage II	611	2.229 (0.939 - 5.293)	0.069	2.055 (0.856 - 4.933)	0.107
Stage III	236	4.968 (2.049 - 12.044)	**< 0.001**	5.278 (2.157 - 12.913)	**< 0.001**
Stage IV	18	26.346 (9.858 - 70.409)	**< 0.001**	30.386 (11.130 - 82.960)	**< 0.001**
Race	977				
Asian	60	Reference			
Black or African American	181	0.912 (0.266 - 3.129)	0.884		
White	736	0.775 (0.242 - 2.483)	0.668		
Histological type	1,058				
Infiltrating Ductal Carcinoma	762	Reference			
Infiltrating Lobular Carcinoma	200	0.469 (0.225 - 0.979)	**0.044**		
Mixed Histology (please specify)	29	1.046 (0.380 - 2.876)	0.931		
Mucinous Carcinoma	16	3.732 (0.904 - 15.410)	0.069		
Metaplastic Carcinoma	9	3.261 (0.450 - 23.657)	0.242		
Other, specify	42	1.186 (0.474 - 2.966)	0.716		
PR status	1,018				
Negative	334	Reference		Reference	
Indeterminate	4	1.337 (0.180 - 9.927)	0.777	1.961 (0.249 - 15.463)	0.523
Positive	680	0.517 (0.333 - 0.805)	**0.003**	0.591 (0.308 - 1.137)	0.115
ER status	1,019				
Negative	232	Reference		Reference	
Indeterminate	2	7.769 (1.045 - 57.757)	**0.045**	4.500 (0.563 - 35.981)	0.156
Positive	785	0.557 (0.349 - 0.888)	**0.014**	0.601 (0.302 - 1.198)	0.148
HER2 status	718				
Negative	552	Reference			
Indeterminate	12	0.000 (0.000 - Inf)	0.997		
Positive	154	1.477 (0.740 - 2.948)	0.269		
JMJD8	1,066				
Low	784	Reference			
High	282	0.55 (0.30 – 0.99)	**0.046**		

HR, hazard ratio; CI, confidence interval.

The bold values indicate statistically significant results.

### 3.5 Correlation between JMJD8 expression and immune infiltration in BRCA

**Figure 4:**
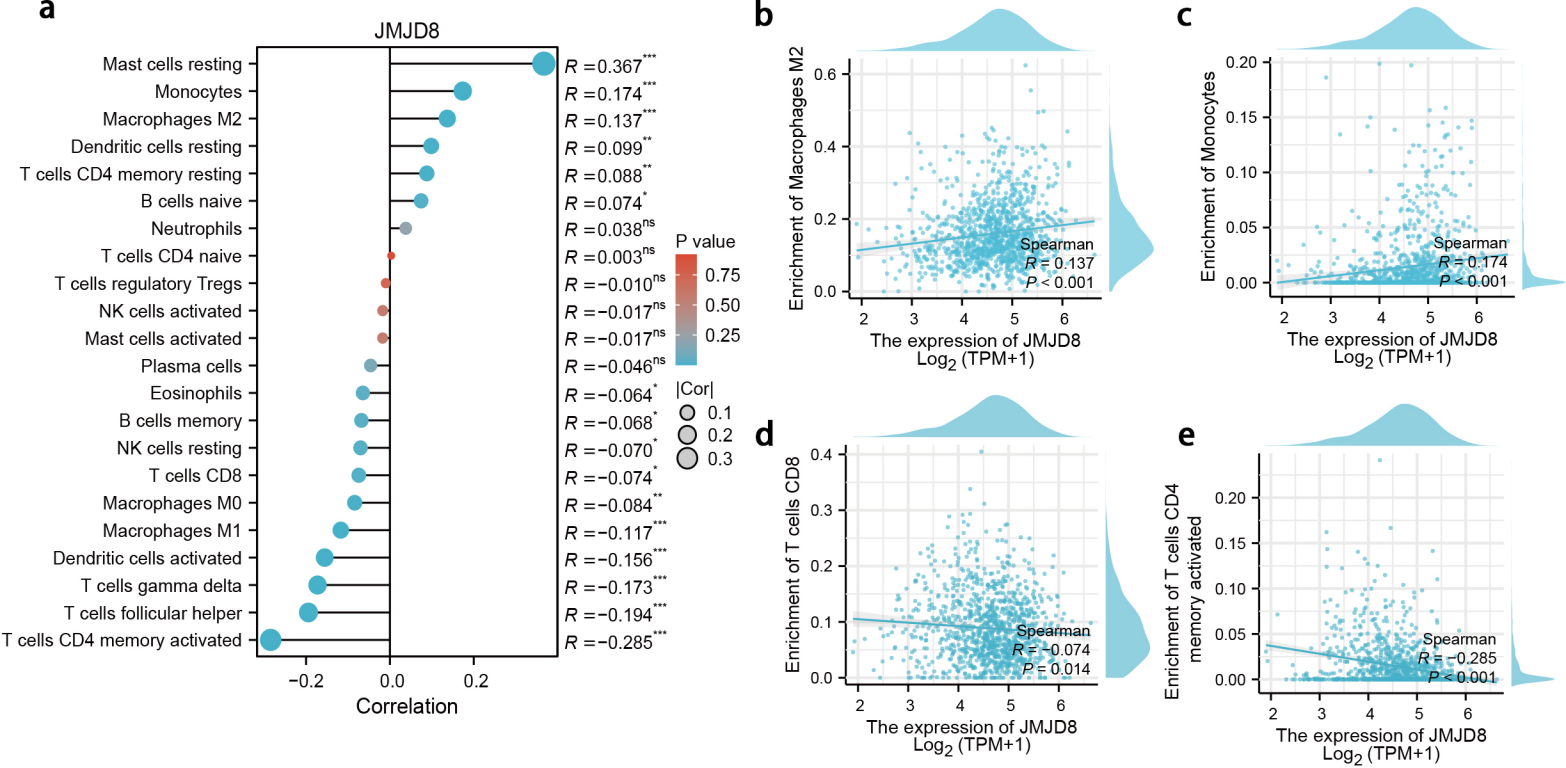
Correlation between JMJD8 expression and immune infiltration in BRCA. **(a)** Landscape of JMJD8 correlations to infiltrating immune cells. *p<0.05; **p<0.01; ***p<0.001; ns, not significant. **(b-e)** Scatter diagram demonstrated the correlation between JMJD8 genes expression and infiltrating immune cells. **(b)** Enrichment of Macrophages M2; **(c)** Enrichment of Monocytes; **(d)** Enrichment of T cells CD8; **(e)** Enrichment of T cells CD4 memory activated cells.

We have characterized the JMJD8-associated immune-infiltrating landscape within the tumor microenvironment of BRCA. Our analysis revealed a positive correlation between JMJD8 expression and the presence of M2 macrophages and monocytes, while demonstrating a negative correlation with CD8 T cells and activated CD4 memory T cells (**Figure 4a**).

These findings suggest that JMJD8 may be linked to immune evasion mechanisms in breast cancer. The positive association with M2 macrophages, which are often implicated in promoting tumor progression and immune suppression, along with the negative association with anti-tumor CD8 T cells, indicates that elevated JMJD8 levels could facilitate an environment conducive to immune escape (**Figures 4b–e**). This highlights the potential role of JMJD8 in modulating the immune landscape of breast cancer, warranting further investigation into its implications for immune response and therapeutic strategies.

### 3.6 Knockdown of JMJD8 activates anti-tumor immune responses to inhibit BRCA proliferation.

**Figure 5:**
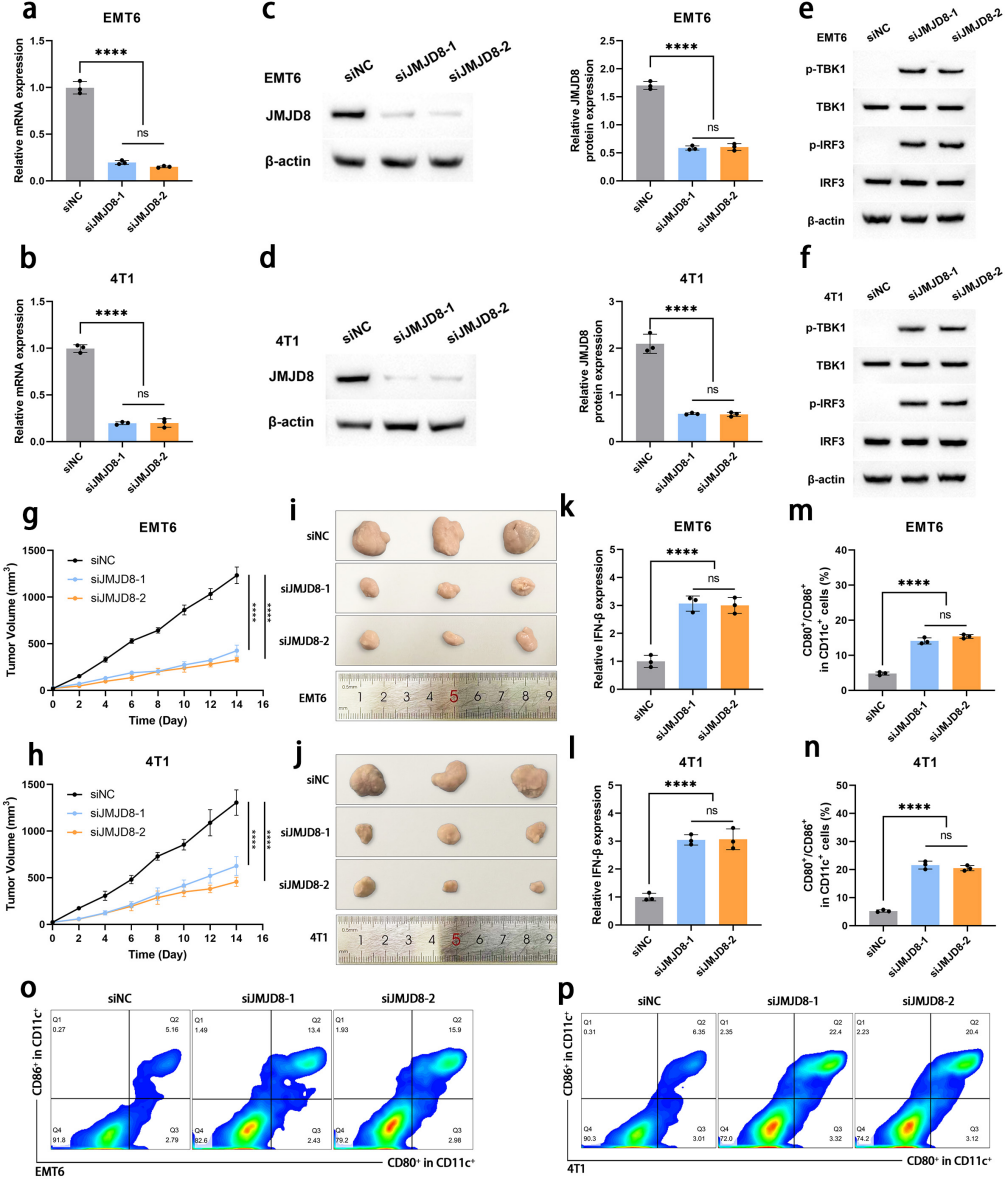
Knockdown of JMJD8 inhibits the proliferation and migration of BRCA cells. Interference efficiency of siJMJD8 in EMT6 and 4T1 was assessed by qRT-PCR **(a, b)** and WB **(c, d)** (n = 3). **(e, f)** Western blotting of p-TBK1, TBK1, p-IRF3, IRF3, and β-actin (n = 3). **(g, h)** Tumor growth curves of EMT6 and 4T1 cells with JMJD8 knocking down (n = 3). **(i, j)** Images of the tumor tissues collected after 14 days (n = 3). **(k, l)** Intertumoral IFN-β·levels in various groups (n = 3). **(m, n)** The proportion of mature DCs (CD80^+^/CD86^+^ gated in CD11c^+^ cells) in tumor-draining lymph nodes detected by flow cytometry (n=3). **(o, p)** Representative flow cytometry images of mature DCs (n = 3). ****p<0.0001; ns, not significant.

To further elucidate the biological role of JMJD8 in the development of BRCA, we employed siRNAs to effectively knock down JMJD8 expression in EMT6 and 4T1 cell lines. The efficiency of this knockdown was confirmed using qRT-PCR (**Figures 5a, b**) and WB analysis (**Figures 5c, d**). Based on this, we assessed the changes in the cGAS-STING pathway following JMJD8 knockdown. It was observed that the knockdown of JMJD8 effectively relieved the inhibition on the phosphorylation of the cGAS-STING pathway (**Figures 5e, f**). This suggests that JMJD8 plays a crucial role in the immune response and tumor progression in breast cancer.

Subsequently, we investigated the impact of JMJD8 knockdown on the proliferative capacity of BRCA cells in an *in vivo* animal model. Our results demonstrated that the depletion of JMJD8 significantly inhibited BRCA cell proliferation (**Figures 5g-j**). To explore the specific effects of JMJD8 on anti-tumor immunity, we collected tumor tissues for immune analysis. IFN-β is a hallmark cytokine that responds to STING activation and plays a critical role in the activation and maturation of antigen-presenting cells (15). ELISA assays indicated that JMJD8 knockout resulted in increased secretion of IFN-β (**Figures 5k, l**). Based on this, we analyzed the migration status of DCs (identified as CCR7^+^ within the CD11c^+^ cells) in the tumor microenvironment. The results showed that the proportion of DCs with migration tendency significantly increased after JMJD8 knockdown, which can be attributed to the enhanced antigen-presenting capacity of DCs mediated by IFN-β signaling (**Supplementary Figure S1**). Additionally, flow cytometric analysis of mature dendritic cells (identified as CD80^+^/CD86^+^ within the CD11c^+^ cells) from tumor-draining lymph nodes revealed that these DCs effectively home to the tumor-draining lymph nodes and undergo maturation (**Figures 5m-p**). We subsequently analyzed the infiltration of CD8^+^ T cells in the tumor microenvironment. Building upon the migration and maturation of DCs, the proportion of CD8^+^ T cell infiltration significantly increased following JMJD8 knockdown (**Supplementary Figure S2**). ELISA results showed that the levels of IL-12 and TNF-α in the tumor microenvironment were upregulated in response to JMJD8 knockdown (**Supplementary Figure S3**). These findings elucidate the impact of JMJD8 on the tumor immunosuppressive microenvironment, demonstrating that its knockdown helps restore anti-tumor immunity in the host.

### 3.7 Relationships between JMJD8 expression and immune markers

To further explore the relationship between JMJD8 and infiltrating immune cells, we conducted a correlation analysis using a coexpression heatmap to assess the associations between JMJD8 and various immune cell markers. These markers are known to characterize immunosuppressive cells, including monocytes and tumor-associated macrophages. The results revealed significant positive correlations between JMJD8 and several key markers: GATA3 (r = 0.517, p < 0.001), STAT6 (r = 0.495, p < 0.001), TNFRSF10C (r = 0.375, p < 0.001), TNFRSF12A (r = 0.175, p < 0.001), BTN2A1 (r = 0.144, p < 0.001), PDCD6 (r = 0.320, p < 0.001), and NECTIN2 (r = 0.529, p < 0.001). This pattern implies that JMJD8 fosters an immune-evasive landscape characterized by suppressive myeloid cells and dampened T cell function.

**Figure 6:**
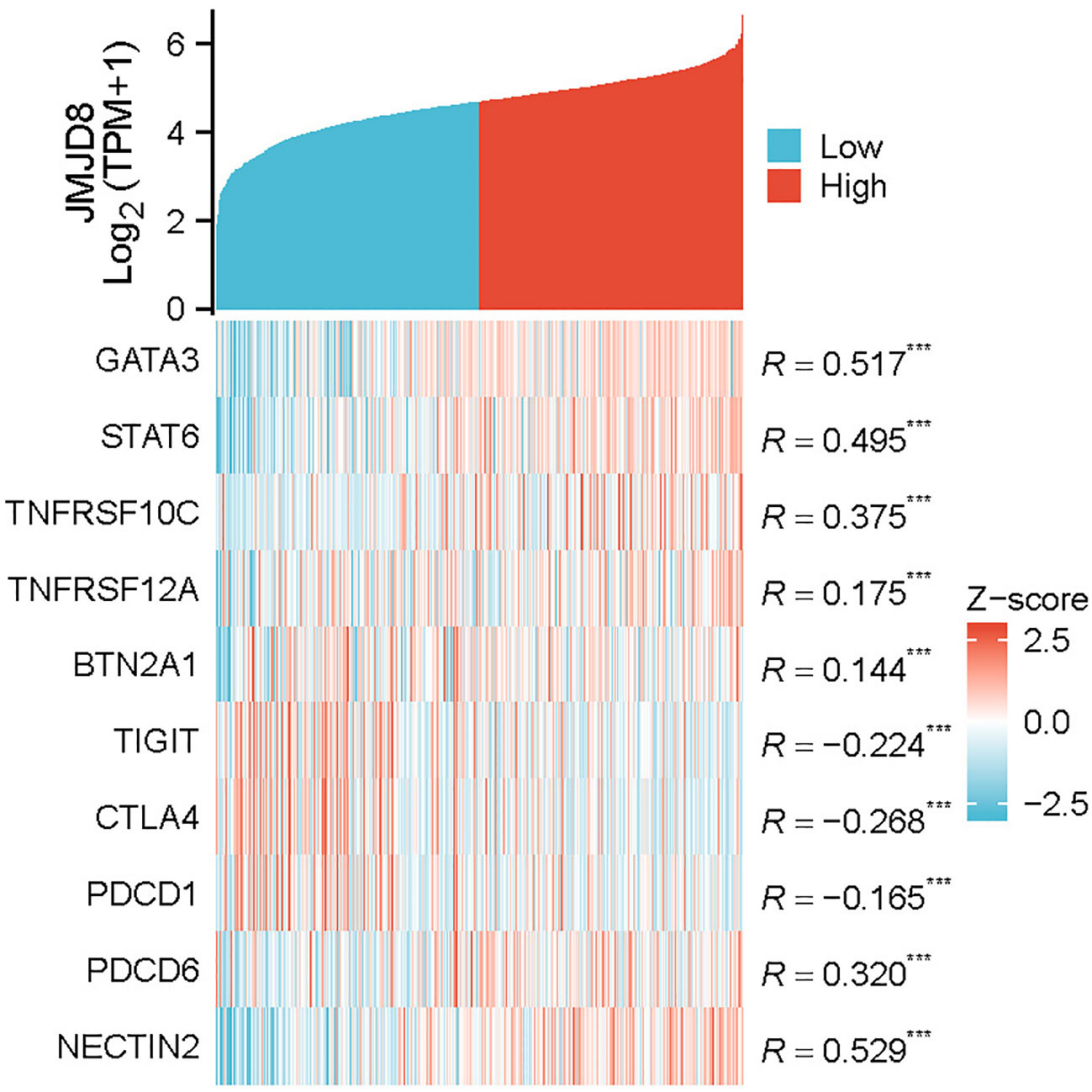
Co-expression heatmap of JMJD8 and infiltrating immune cell markers. ***p<0.001.

In contrast, we also examined the correlation of JMJD8 with T cell markers, including PDCD1 (r = -0.165, p < 0.001), CTLA4 (r = -0.268, p < 0.001), and TIGIT (r = -0.224, p < 0.001) (**Figure 6**). Notably, these results suggest that JMJD8 is negatively correlated with immunosuppressive immune checkpoints. Critically, functional knockdown experiments demonstrate that reducing JMJD8 expression leads to a significant upregulation of PD-L1(**Supplementary Figure S4**). This compensatory PD-L1 induction reveals a vulnerability: Tumors with high JMJD8 expression may exhibit reduced sensitivity to immune checkpoint inhibitors (ICIs) targeting the PD-1/PD-L1 axis due to the dominance of its immunosuppressive myeloid programs. This suggests that combinatorial approaches involving JMJD8 inhibition (e.g., siRNA, small molecules) and anti-PD-1/PD-L1 immune checkpoint blockade could be particularly effective in overcoming JMJD8-mediated immunosuppression and enhancing ICI response rates.

## 4 Discussion

As one of the most common cancers affecting women worldwide, breast cancer is characterized by persistently high incidence and mortality rates (16, 17). To effectively progress and metastasize, breast tumors engage in a dynamic process of remodeling the surrounding tissues, thereby establishing a microenvironment that supports their growth and dissemination (18). This remodeling involves significant alterations in the extracellular matrix and the vascular system, along with changes in various supporting cells. Key components of this supportive network include stromal cells, such as fibroblasts and adipocytes, as well as immune cells and endothelial cells. Collectively, these alterations facilitate tumor progression by creating a niche that enhances tumor survival and invasiveness (19). Unfortunately, the complexity of this immunosuppressive microenvironment often limits the effectiveness of clinical treatment regimens, posing significant challenges for successful therapeutic outcomes in breast cancer patients (14). The intricate heterogeneity of the immune microenvironment in breast cancer, along with the challenges encountered by current tumor immunotherapies, highlights the necessity for a deeper understanding of these complex interactions.

A growing body of research has elucidated the close association between proteins containing the JmjC domain and cancer development. These proteins are not only implicated in the processes of tumor proliferation but also exert substantial effects on the tumor microenvironment, highlighting their multifaceted roles in cancer biology. JMJD8, as a distinctive member of the JMJD family, has lost its demethylation capacity; however, it remains intricately involved in a range of physiological functions (6). This duality underscores its significant role in cellular processes, despite the loss of a key enzymatic activity typically associated with this family of proteins. While existing research has characterized aberrant JMJD8 overexpression in tumor tissues and its prognostic relevance across various malignancies, such investigations have predominantly centered on delineating JMJD8’s molecular features and phenotypic correlations. Nevertheless, the precise mechanisms underlying JMJD8’s involvement in BRCA progression, particularly its functional significance in modulating tumor immune responses, remain insufficiently understood and warrant systematic exploration (9–11). This gap in understanding underscores the need for further investigation into JMJD8’s specific functions within the context of BRCA and its potential implications for tumor immunity. Our research shows that JMJD8 is highly expressed in BRCA compared to normal tissue and is associated with various indicators of BRCA. Importantly, multivariate Cox analysis indicated that JMJD8 expression serves as an independent factor influencing overall survival in BRCA patients. ROC analysis confirmed the potential prognostic value of JMJD8, and Kaplan-Meier survival analysis revealed that patients with high JMJD8 expression experienced significantly shorter overall survival.

The cGAS-STING pathway serves as a crucial mechanism for immune cells to detect tumor-specific antigens. This pathway is initiated when cyclic GMP-AMP (cGAMP) synthase (cGAS) recognizes DNA released from dying tumor cells (20). This recognition triggers a cascade of phosphorylation events involving STING, TBK1, and IRF3, ultimately leading to the secretion of type I interferons, which induce an anti-tumor immune response (21, 22). The phosphorylation activation of the cGAS-STING pathway initiates a complex type I interferon-driven inflammatory response, enhancing the activation of DCs and facilitating the cross-presentation of tumor antigens, thereby supporting the subsequent initial activation of T cells (23, 24). Previous studies have indicated that JMJD8 may interfere with the cGAS-STING pathway and the tumor microenvironment (9, 11). To further investigate the relationship between JMJD8 and the progression of BRCA as well as immune responses, we established JMJD8 knockout breast cancer cell lines for both cellular and animal studies. Our findings indicate that the knockdown of JMJD8 significantly upregulates the overall phosphorylation activation of the cGAS-STING pathway. Subsequently, in breast cancer animal models, JMJD8 knockdown significantly inhibited the proliferation of breast cancer cells, resulting in reduced tumor size and weight, along with an upregulation of IFN-β. This discovery suggests that the absence of JMJD8 alleviates the inhibition of the cGAS-STING pathway, thereby activating antitumor immunity and restricting tumor proliferation.

After being induced by IFN-β to infiltrate the tumor microenvironment, DCs phagocytose and process tumor-specific antigens. They then need to return to the tumor-draining lymph nodes, where they differentiate into mature DCs to present antigens to other lymphocytes, thereby initiating an anti-tumor immune response (25, 26). The successful maturation of DCs signifies an improvement in the tumor microenvironment. We assessed the functionality of DCs following JMJD8 knockdown using flow cytometry. Notably, JMJD8 knockdown was found to promote the migration and maturation of DCs. This indicates that high expression levels of JMJD8 exacerbate the immunosuppressive tumor microenvironment, hindering antigen presentation and the initiation of anti-tumor immune responses. These findings suggest that JMJD8 influences the prognosis of BRCA patients by modulating the tumor immune microenvironment.

To delineate the role of JMJD8 in sculpting the tumor immune microenvironment, we established its co-expression profile with immunosuppressive infiltrates, revealing significant positive correlations with monocyte/tumor-associated macrophage markers that drive myeloid-driven immune evasion. Conversely, JMJD8 exhibited negative correlations with T-cell exhaustion checkpoints, suppressing cytotoxic responses. Critically, functional validation demonstrated JMJD8 knockdown triggers compensatory PD-L1 upregulation, mechanistically explaining reduced sensitivity to PD-1/PD-L1 inhibitors in JMJD8-high tumors. These findings position JMJD8 as a dual-axis regulator of immunosuppression and reveal its targeting combined with anti-PD-1/PD-L1 blockade as a rational strategy to reverse immune evasion and enhance therapeutic efficacy in breast cancer models.

In conclusion, this study demonstrates that JMJD8 expression can serve not only as a diagnostic factor for the progression of BRCA but also as an independent prognostic marker. The correlation analysis of JMJD8 with various clinical and pathological parameters further supports this hypothesis. High JMJD8 expression promotes the proliferation of BRCA cells and is significantly associated with poor prognosis. Furthermore, our research indicates that JMJD8 gene expression is diversely linked to the immunosuppressive microenvironment in BRCA, highlighting its potential as a therapeutic target for immunotherapy in this context.

## Data Availability

The datasets presented in this study can be found in online repositories. The names of the repository/repositories and accession number(s) can be found below: https://www.ncbi.nlm.nih.gov/, PHS000178.
