# Supplementary material for: JMJD8 overexpression in breast cancer: implications for diagnosis, prognosis, and immune microenvironment interactions
Source: Front Oncol. 2025 Jul 21;15:1536278. doi: 10.3389/fonc.2025.1536278 (PMC12318979; doi:10.3389/fonc.2025.1536278)
Supplement: Supplementary file 1 [file DataSheet1.docx]

**JMJD8 Overexpression in Breast Cancer: Implications for Diagnosis, Prognosis, and Immune Microenvironment Interactions**

**Chenlei Zhu^1†^, Tianyi Xi^1†^, Guorong Yang^1^, Wen Lu^1^, Sentai Wang^1*^, Jiwei Cao^1*^**

^1^Department of General Surgery, The Affiliated Suzhou Hospital of Nanjing Medical University, Suzhou, 215000, China

*Correspondence:

Jiwei Cao

[caojiwei2024@126.com](mailto:caojiwei2024@126.com)

^†^Chenlei Zhu and Tianyi Xi contributed equally to this paper.

# Supplementary Materials

**Table S1. Expression of the JMJD8 in BRCA**

**Figure S1.** **Flow cytometry analysis of DC migration in tumor tissue.**

**Figure S2. Flow cytometry analysis of CD8^+^ T cell infiltration in tumor tissue.**

**Figure S3. ELISA analysis of IL-12 and TNF-α in the tumor microenvironment.**

**Figure S4. Western blot of PD-L1 expression in tumor tissues.**

**Table S1. Expression of the JMJD8 in BRCA**

| pair_info | status | JMJD8 |
| --- | --- | --- |
| TCGA-A7-A0CE | Tumor | 3.49675633 |
| TCGA-A7-A0CE | Normal | 4.47545459 |
| TCGA-A7-A0CH | Tumor | 4.97674855 |
| TCGA-A7-A0CH | Normal | 4.36143852 |
| TCGA-A7-A0D9 | Tumor | 4.25903892 |
| TCGA-A7-A0D9 | Normal | 3.98335848 |
| TCGA-A7-A0DB | Tumor | 4.96067851 |
| TCGA-A7-A0DB | Normal | 4.08568808 |
| TCGA-A7-A0DC | Tumor | 3.01031438 |
| TCGA-A7-A0DC | Normal | 4.49307086 |
| TCGA-A7-A13E | Tumor | 5.01592123 |
| TCGA-A7-A13E | Normal | 4.40631897 |
| TCGA-A7-A13F | Tumor | 4.98890278 |
| TCGA-A7-A13F | Normal | 4.13523113 |
| TCGA-A7-A13G | Tumor | 3.99562923 |
| TCGA-A7-A13G | Normal | 3.36889404 |
| TCGA-AC-A23H | Tumor | 4.52265842 |
| TCGA-AC-A23H | Normal | 4.02332633 |
| TCGA-AC-A2FB | Tumor | 4.72643344 |
| TCGA-AC-A2FB | Normal | 4.65265284 |
| TCGA-AC-A2FF | Tumor | 4.71627211 |
| TCGA-AC-A2FF | Normal | 4.63882106 |
| TCGA-AC-A2FM | Tumor | 5.28309947 |
| TCGA-AC-A2FM | Normal | 4.59067387 |
| TCGA-BH-A0AU | Tumor | 5.72229114 |
| TCGA-BH-A0AU | Normal | 4.54933649 |
| TCGA-BH-A0AY | Tumor | 4.94331187 |
| TCGA-BH-A0AY | Normal | 4.40820234 |
| TCGA-BH-A0AZ | Tumor | 4.31816497 |
| TCGA-BH-A0AZ | Normal | 4.42977663 |
| TCGA-BH-A0B3 | Tumor | 4.13203388 |
| TCGA-BH-A0B3 | Normal | 4.43014474 |
| TCGA-BH-A0B5 | Tumor | 5.34845552 |
| TCGA-BH-A0B5 | Normal | 3.76773954 |
| TCGA-BH-A0B7 | Tumor | 4.38474749 |
| TCGA-BH-A0B7 | Normal | 4.17696099 |
| TCGA-BH-A0B8 | Tumor | 4.5766612 |
| TCGA-BH-A0B8 | Normal | 3.88664769 |
| TCGA-BH-A0BA | Tumor | 5.30968916 |
| TCGA-BH-A0BA | Normal | 4.67498033 |
| TCGA-BH-A0BC | Tumor | 4.82637561 |
| TCGA-BH-A0BC | Normal | 4.26225273 |
| TCGA-BH-A0BJ | Tumor | 4.66760265 |
| TCGA-BH-A0BJ | Normal | 4.53450988 |
| TCGA-BH-A0BM | Tumor | 3.63665942 |
| TCGA-BH-A0BM | Normal | 4.39085338 |
| TCGA-BH-A0BQ | Tumor | 5.21985001 |
| TCGA-BH-A0BQ | Normal | 4.45153424 |
| TCGA-BH-A0BS | Tumor | 4.92960166 |
| TCGA-BH-A0BS | Normal | 4.7346175 |
| TCGA-BH-A0BT | Tumor | 5.40207066 |
| TCGA-BH-A0BT | Normal | 4.77774057 |
| TCGA-BH-A0BV | Tumor | 4.57051758 |
| TCGA-BH-A0BV | Normal | 4.37533142 |
| TCGA-BH-A0BW | Tumor | 4.21448233 |
| TCGA-BH-A0BW | Normal | 3.62132516 |
| TCGA-BH-A0BZ | Tumor | 4.8854133 |
| TCGA-BH-A0BZ | Normal | 4.37392656 |
| TCGA-BH-A0C0 | Tumor | 4.68048752 |
| TCGA-BH-A0C0 | Normal | 4.54589426 |
| TCGA-BH-A0C3 | Tumor | 5.38971823 |
| TCGA-BH-A0C3 | Normal | 4.51482292 |
| TCGA-BH-A0DD | Tumor | 4.65825719 |
| TCGA-BH-A0DD | Normal | 4.38007139 |
| TCGA-BH-A0DG | Tumor | 4.64259761 |
| TCGA-BH-A0DG | Normal | 4.1890101 |
| TCGA-BH-A0DH | Tumor | 4.99905743 |
| TCGA-BH-A0DH | Normal | 4.18195328 |
| TCGA-BH-A0DK | Tumor | 4.06249305 |
| TCGA-BH-A0DK | Normal | 4.72658058 |
| TCGA-BH-A0DL | Tumor | 3.82473731 |
| TCGA-BH-A0DL | Normal | 4.30553283 |
| TCGA-BH-A0DO | Tumor | 5.23515262 |
| TCGA-BH-A0DO | Normal | 4.53262242 |
| TCGA-BH-A0DP | Tumor | 5.24354723 |
| TCGA-BH-A0DP | Normal | 4.82558722 |
| TCGA-BH-A0DQ | Tumor | 4.50788976 |
| TCGA-BH-A0DQ | Normal | 4.6547334 |
| TCGA-BH-A0DT | Tumor | 5.24066542 |
| TCGA-BH-A0DT | Normal | 4.5334263 |
| TCGA-BH-A0DV | Tumor | 5.18328006 |
| TCGA-BH-A0DV | Normal | 4.47470859 |
| TCGA-BH-A0DZ | Tumor | 4.38696955 |
| TCGA-BH-A0DZ | Normal | 4.99554331 |
| TCGA-BH-A0E0 | Tumor | 3.76899942 |
| TCGA-BH-A0E0 | Normal | 4.585245 |
| TCGA-BH-A0E1 | Tumor | 5.15564805 |
| TCGA-BH-A0E1 | Normal | 4.83680276 |
| TCGA-BH-A0H5 | Tumor | 4.23013383 |
| TCGA-BH-A0H5 | Normal | 4.28600876 |
| TCGA-BH-A0H7 | Tumor | 4.24179443 |
| TCGA-BH-A0H7 | Normal | 4.52097533 |
| TCGA-BH-A0H9 | Tumor | 5.51885927 |
| TCGA-BH-A0H9 | Normal | 4.25436728 |
| TCGA-BH-A0HA | Tumor | 4.14340158 |
| TCGA-BH-A0HA | Normal | 4.5316497 |
| TCGA-BH-A0HK | Tumor | 4.02403594 |
| TCGA-BH-A0HK | Normal | 4.82377993 |
| TCGA-BH-A18J | Tumor | 4.62394293 |
| TCGA-BH-A18J | Normal | 4.56238898 |
| TCGA-BH-A18K | Tumor | 4.67608119 |
| TCGA-BH-A18K | Normal | 4.51794963 |
| TCGA-BH-A18L | Tumor | 5.40925854 |
| TCGA-BH-A18L | Normal | 4.54036211 |
| TCGA-BH-A18M | Tumor | 5.24420588 |
| TCGA-BH-A18M | Normal | 4.62243258 |
| TCGA-BH-A18N | Tumor | 6.09907634 |
| TCGA-BH-A18N | Normal | 4.42280485 |
| TCGA-BH-A18P | Tumor | 4.65452147 |
| TCGA-BH-A18P | Normal | 3.89745343 |
| TCGA-BH-A18Q | Tumor | 5.09698285 |
| TCGA-BH-A18Q | Normal | 4.51612234 |
| TCGA-BH-A18R | Tumor | 4.10814805 |
| TCGA-BH-A18R | Normal | 4.03453268 |
| TCGA-BH-A18S | Tumor | 4.6272683 |
| TCGA-BH-A18S | Normal | 4.35378906 |
| TCGA-BH-A18U | Tumor | 4.81036084 |
| TCGA-BH-A18U | Normal | 4.8972259 |
| TCGA-BH-A18V | Tumor | 4.482887 |
| TCGA-BH-A18V | Normal | 3.89545774 |
| TCGA-BH-A1EN | Tumor | 4.61841487 |
| TCGA-BH-A1EN | Normal | 4.04483123 |
| TCGA-BH-A1EO | Tumor | 4.5518728 |
| TCGA-BH-A1EO | Normal | 4.04118164 |
| TCGA-BH-A1ET | Tumor | 5.3202536 |
| TCGA-BH-A1ET | Normal | 4.27783601 |
| TCGA-BH-A1EU | Tumor | 4.59722723 |
| TCGA-BH-A1EU | Normal | 4.01571165 |
| TCGA-BH-A1EV | Tumor | 4.93600655 |
| TCGA-BH-A1EV | Normal | 3.8543448 |
| TCGA-BH-A1EW | Tumor | 4.94546256 |
| TCGA-BH-A1EW | Normal | 4.24858038 |
| TCGA-BH-A1F0 | Tumor | 3.51571245 |
| TCGA-BH-A1F0 | Normal | 4.17196737 |
| TCGA-BH-A1F2 | Tumor | 4.13248636 |
| TCGA-BH-A1F2 | Normal | 3.90459007 |
| TCGA-BH-A1F6 | Tumor | 4.35546034 |
| TCGA-BH-A1F6 | Normal | 3.91928287 |
| TCGA-BH-A1F8 | Tumor | 4.25279409 |
| TCGA-BH-A1F8 | Normal | 4.44332153 |
| TCGA-BH-A1FB | Tumor | 5.07235023 |
| TCGA-BH-A1FB | Normal | 4.25309676 |
| TCGA-BH-A1FC | Tumor | 3.14068051 |
| TCGA-BH-A1FC | Normal | 4.15106323 |
| TCGA-BH-A1FD | Tumor | 4.73434653 |
| TCGA-BH-A1FD | Normal | 4.02940871 |
| TCGA-BH-A1FE | Tumor | 4.39648148 |
| TCGA-BH-A1FE | Normal | 3.56626719 |
| TCGA-BH-A1FG | Tumor | 5.20875524 |
| TCGA-BH-A1FG | Normal | 2.76805726 |
| TCGA-BH-A1FH | Tumor | 4.53874921 |
| TCGA-BH-A1FH | Normal | 4.44777754 |
| TCGA-BH-A1FJ | Tumor | 4.59152983 |
| TCGA-BH-A1FJ | Normal | 3.81582132 |
| TCGA-BH-A1FM | Tumor | 3.70313393 |
| TCGA-BH-A1FM | Normal | 4.29613432 |
| TCGA-BH-A1FN | Tumor | 3.82508344 |
| TCGA-BH-A1FN | Normal | 3.80940415 |
| TCGA-BH-A1FR | Tumor | 4.32228872 |
| TCGA-BH-A1FR | Normal | 4.09039617 |
| TCGA-BH-A1FU | Tumor | 4.05088474 |
| TCGA-BH-A1FU | Normal | 4.74281467 |
| TCGA-BH-A203 | Tumor | 3.30818611 |
| TCGA-BH-A203 | Normal | 3.97581358 |
| TCGA-BH-A204 | Tumor | 4.92225019 |
| TCGA-BH-A204 | Normal | 3.63860678 |
| TCGA-BH-A208 | Tumor | 4.22077879 |
| TCGA-BH-A208 | Normal | 4.25415561 |
| TCGA-BH-A209 | Tumor | 3.33196312 |
| TCGA-BH-A209 | Normal | 4.11660596 |
| TCGA-E2-A153 | Tumor | 5.24335681 |
| TCGA-E2-A153 | Normal | 4.76178524 |
| TCGA-E2-A158 | Tumor | 3.47257875 |
| TCGA-E2-A158 | Normal | 4.25355821 |
| TCGA-E2-A15I | Tumor | 6.09717885 |
| TCGA-E2-A15I | Normal | 3.60834798 |
| TCGA-E2-A15K | Tumor | 5.79615385 |
| TCGA-E2-A15K | Normal | 4.37033868 |
| TCGA-E2-A15M | Tumor | 3.90199638 |
| TCGA-E2-A15M | Normal | 4.28205424 |
| TCGA-E2-A1BC | Tumor | 5.42015561 |
| TCGA-E2-A1BC | Normal | 3.8980632 |
| TCGA-E2-A1IG | Tumor | 4.52955237 |
| TCGA-E2-A1IG | Normal | 4.33748275 |
| TCGA-E2-A1L7 | Tumor | 4.27895118 |
| TCGA-E2-A1L7 | Normal | 4.67157079 |
| TCGA-E2-A1LB | Tumor | 4.45252296 |
| TCGA-E2-A1LB | Normal | 4.5341301 |
| TCGA-E2-A1LH | Tumor | 3.24432387 |
| TCGA-E2-A1LH | Normal | 4.67868041 |
| TCGA-E2-A1LS | Tumor | 2.71784638 |
| TCGA-E2-A1LS | Normal | 3.58807296 |
| TCGA-E9-A1N4 | Tumor | 4.44131083 |
| TCGA-E9-A1N4 | Normal | 4.29284063 |
| TCGA-E9-A1N5 | Tumor | 5.8769859 |
| TCGA-E9-A1N5 | Normal | 4.71652455 |
| TCGA-E9-A1N6 | Tumor | 5.01044866 |
| TCGA-E9-A1N6 | Normal | 4.21381385 |
| TCGA-E9-A1N9 | Tumor | 3.87782276 |
| TCGA-E9-A1N9 | Normal | 4.62302403 |
| TCGA-E9-A1NA | Tumor | 4.81476061 |
| TCGA-E9-A1NA | Normal | 4.69352041 |
| TCGA-E9-A1ND | Tumor | 3.94450241 |
| TCGA-E9-A1ND | Normal | 4.29502501 |
| TCGA-E9-A1NF | Tumor | 5.15077899 |
| TCGA-E9-A1NF | Normal | 4.57613511 |
| TCGA-E9-A1NG | Tumor | 4.78799656 |
| TCGA-E9-A1NG | Normal | 4.11278352 |
| TCGA-E9-A1R7 | Tumor | 4.36618231 |
| TCGA-E9-A1R7 | Normal | 4.12928302 |
| TCGA-E9-A1RB | Tumor | 5.5770088 |
| TCGA-E9-A1RB | Normal | 4.20419551 |
| TCGA-E9-A1RC | Tumor | 6.21522429 |
| TCGA-E9-A1RC | Normal | 4.06365825 |
| TCGA-E9-A1RD | Tumor | 4.69568765 |
| TCGA-E9-A1RD | Normal | 4.21088755 |
| TCGA-E9-A1RF | Tumor | 3.98505407 |
| TCGA-E9-A1RF | Normal | 3.59017084 |
| TCGA-E9-A1RH | Tumor | 4.32920972 |
| TCGA-E9-A1RH | Normal | 3.56282854 |
| TCGA-E9-A1RI | Tumor | 4.77074449 |
| TCGA-E9-A1RI | Normal | 3.7761777 |
| TCGA-GI-A2C8 | Tumor | 5.11325876 |
| TCGA-GI-A2C8 | Normal | 2.45220005 |
| TCGA-GI-A2C9 | Tumor | 3.9046575 |
| TCGA-GI-A2C9 | Normal | 4.03654729 |

**Figure S1.** **Flow cytometry analysis of DC migration in tumor tissue.** (a) Representative flow cytometric imaging of CCR7^+^ in CD11c^+^ cells infiltrated in EMT6 tumor tissues. (b) Quantification of CCR7^+^ in CD11c^+^ cells infiltrated in EMT6 tumor tissues (n = 3). (c) Representative flow cytometric imaging of CCR7^+^ in CD11c^+^ cells infiltrated in 4T1 tumor tissues. (d) Quantification of CCR7^+^ in CD11c^+^ cells infiltrated in 4T1 tumor tissues (n = 3).

**Figure S2. Flow cytometry analysis of CD8^+^ T cell infiltration in tumor tissue.** (a) Representative flow cytometric imaging of CD8^+^ in CD3^+^ T cells infiltrated in EMT6 tumor tissues. (b) Quantification of CD8^+^ in CD3^+^ T cells infiltrated in EMT6 tumor tissues (n = 3). (c) Representative flow cytometric imaging of CD8^+^ in CD3^+^ T cells infiltrated in 4T1 tumor tissues. (d) Quantification of CD8^+^ in CD3^+^ T cells infiltrated in 4T1 tumor tissues (n = 3).

**Figure S3. ELISA analysis of IL-12 and TNF-α in the tumor microenvironment.** IL-12 expression in EMT6 (a) and 4T1 (b) tumor microenvironment (n = 3). TNF-α expression in EMT6 (c) and 4T1 (d) tumor microenvironment (n = 3).

**Figure S4. Western blot of PD-L1 expression in tumor tissues after various treatments.**
