# Supplementary figures and images for: JMJD8 overexpression in breast cancer: implications for diagnosis, prognosis, and immune microenvironment interactions
Source: Front Oncol. 2025 Jul 21;15:1536278. doi: 10.3389/fonc.2025.1536278 (PMC12318979; doi:10.3389/fonc.2025.1536278)

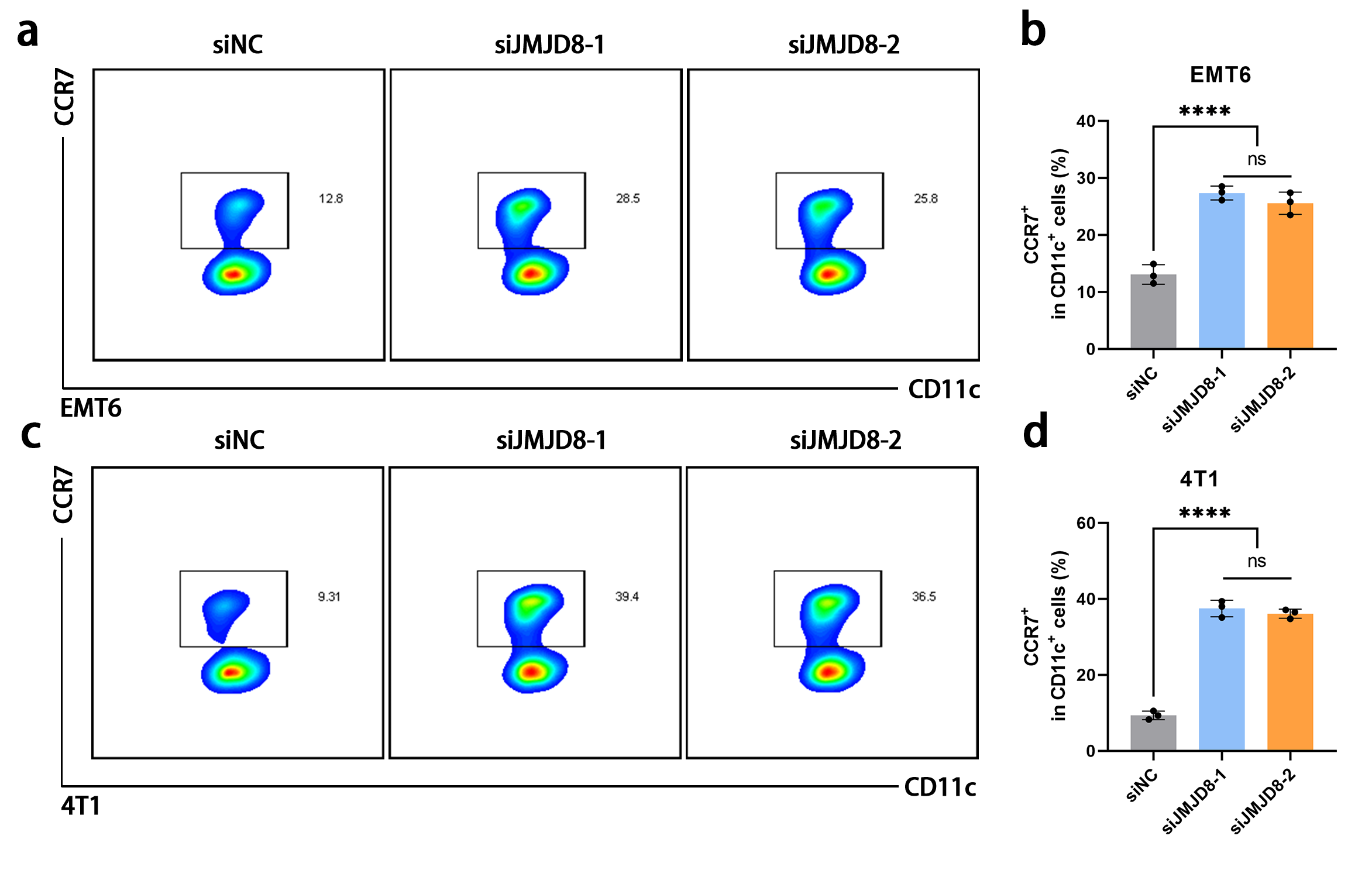

Supplement: Supplementary file 2 [file Image1.png]

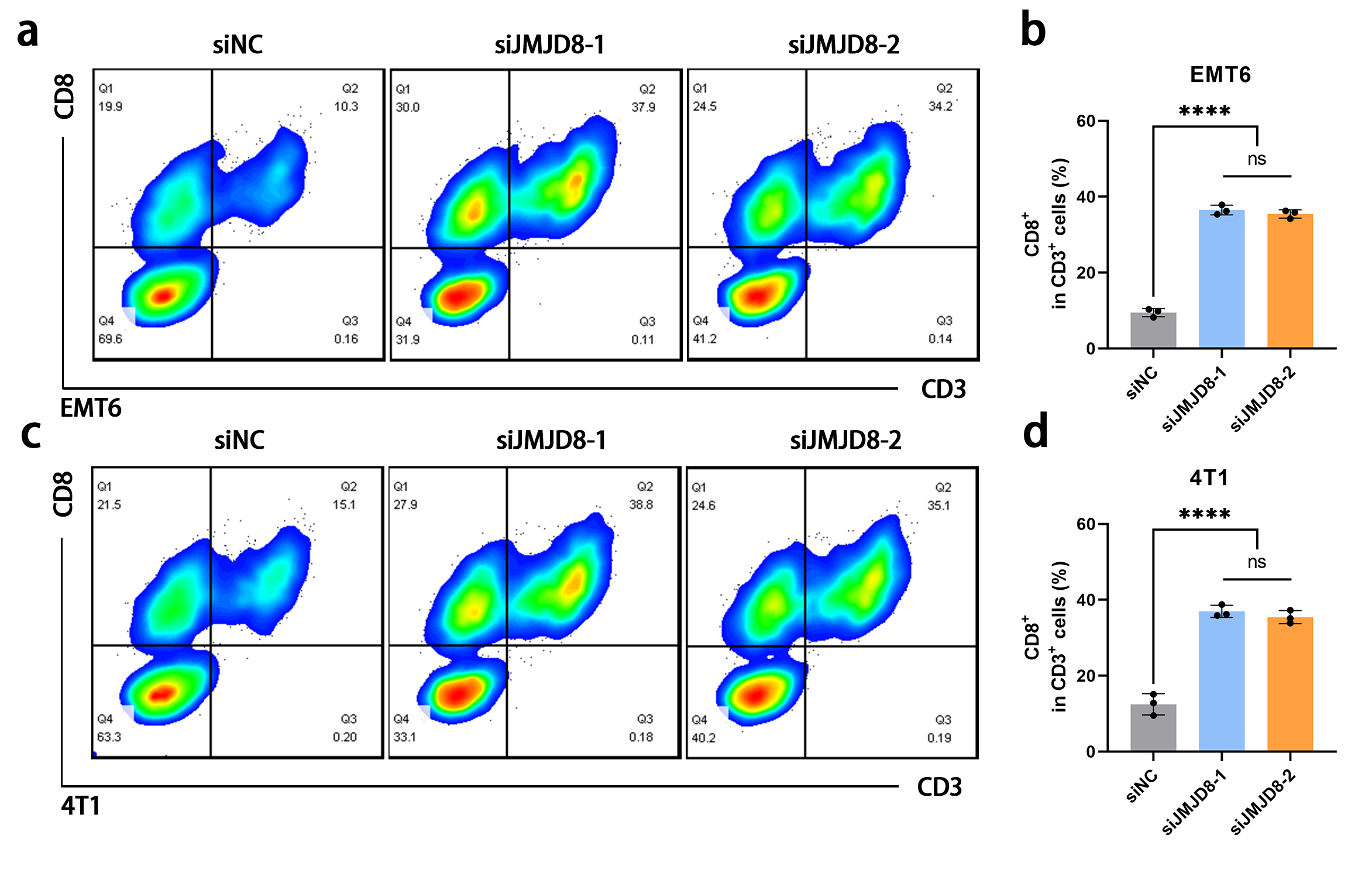

Supplement: Supplementary file 3 [file Image2.png]

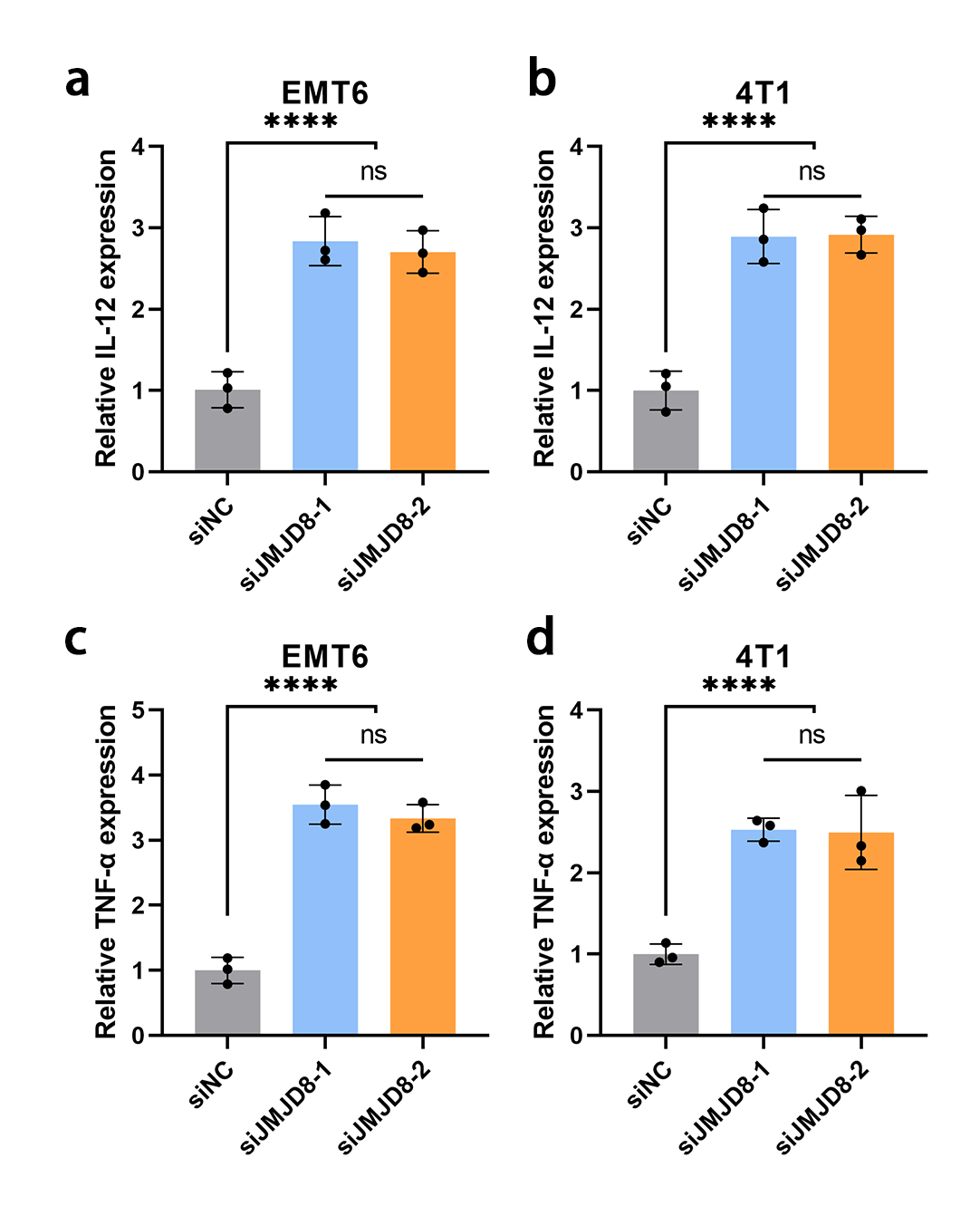

Supplement: Supplementary file 4 [file Image3.png]

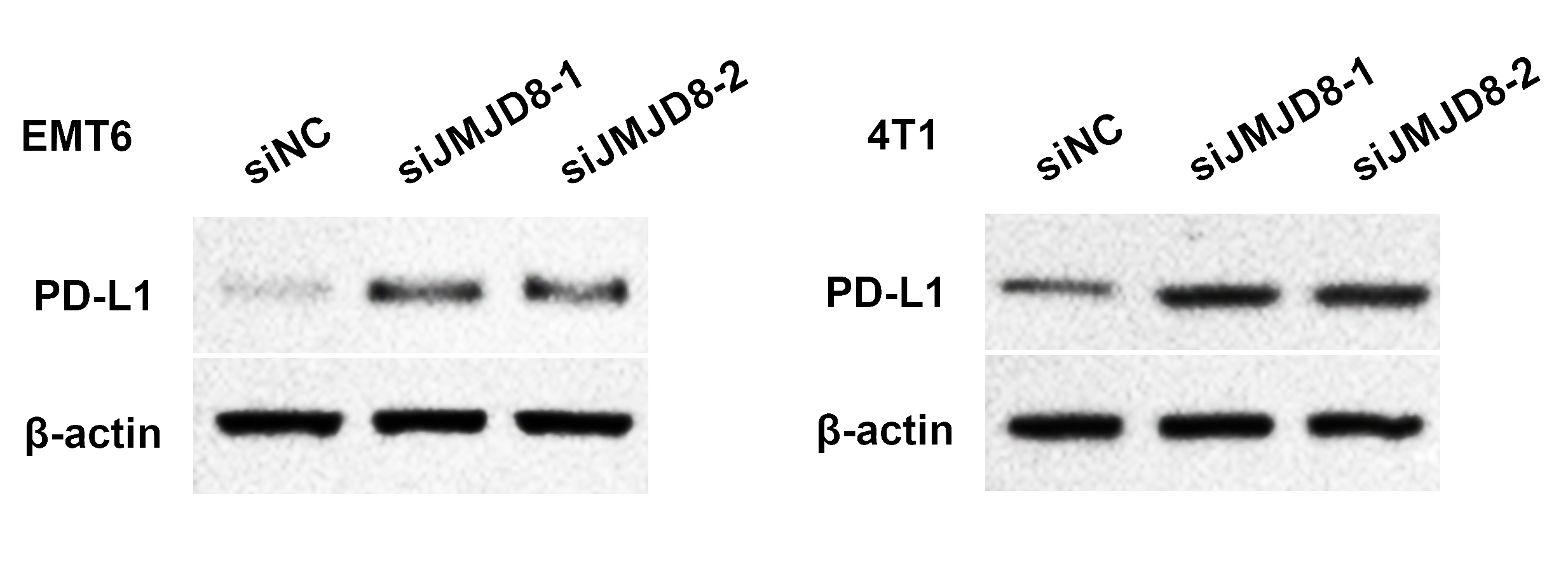

Supplement: Supplementary file 5 [file Image4.png]
